# Neurodegeneration Focused Biorepositories: NCRAD, BioSEND, MJFF

**DOI:** 10.1002/alz.091931

**Published:** 2025-01-09

**Authors:** Michael C. Edler, Kaci Lacy, Casey Snoddy, Colleen M. Mitchell, K. Rose Case, Chris Hobbick, Kelly N. Nudelman, Jan Hamer, Claire Wegel, Kelley M. Faber, Jacqueline M Jackson, Tatiana M. Foroud

**Affiliations:** ^1^ Department of Medical and Molecular Genetics, Indiana University School of Medicine, Indianapolis, IN USA; ^2^ National Centralized Repository for Alzheimer's Disease and Related Dementias (NCRAD), Indianapolis, IN USA; ^3^ Indiana Alzheimer's Disease Research Center, Indianapolis, IN USA

## Abstract

**Background:**

Biorepositories play an integral role in the advancement of our understanding of neurodegenerative diseases and improving human health outcomes. Research efforts are accelerated when access to high‐quality clinical specimens is made available from a large, diverse participant group. Indiana University is home to three important neurodegenerative disease‐focused biorepositories including the NIA‐funded National Centralized Repository for Alzheimer’s Disease and Related Dementias (NCRAD), the NINDS‐funded Biospecimen Exchange for Neurological Disorders (BioSEND), and the Michael J. Fox Foundation (MJFF) biorepository. Having all three repositories in one location presents a unique opportunity to leverage common protocols and shared resources to provide researchers with access to a wide breadth of specimens, facilitating broad specimen sharing and standardization across studies and repositories.

**Method:**

All three repositories provide exceptional data and study coordination, data management, scientific support, and technical resources. Uniform best practice standard operating procedures (SOPs) and materials for collection and processing of samples yield consistently high‐quality specimens across studies from all repositories. Banked specimen types include DNA, RNA, whole blood, plasma, serum, cerebrospinal fluid, urine, stool, brain tissue, peripheral blood mononuclear cells (PBMCs), fibroblasts, and induced pluripotent stem cells (iPSCs). Online catalogs are available for specimen selection along with project management that can assist in the selection and approval of specimens across studies and repositories.

**Result:**

Among the three repositories, over 4 million specimen aliquots from more than 150 studies have been banked. This collection represents over 142,000 unique participants across a diverse array of neurodegenerative diseases, including Alzheimer’s disease, Parkinson’s disease, Amyotrophic Lateral Sclerosis, Frontotemporal Dementia, Lewy Body Dementia, Concussion, Traumatic Brain Injury, Huntington’s Disease, Spinocerebellar Ataxia and other neurodegenerative proteinopathies. More than 600,000 specimen aliquots have been distributed to over 370 investigators, resulting in almost 1,000 publications.

**Conclusion:**

NCRAD, BioSEND, and MJFF serve as crucial resources for investigators requiring high‐quality clinical specimens collected and processed in accordance with best practice SOPs. The centralized location at Indiana University provides the opportunity to synergize and align common study objectives to provide shared resources with broader sample diversity, increased statistical power, improved reproducibility, and enhanced opportunities for broad specimen sharing, thereby fostering collaborative research initiatives.

